# Commentary: Gut Microbiome and Space Travelers' Health: State of the Art and Possible Pro/Prebiotic Strategies for Long-Term Space Missions

**DOI:** 10.3389/fphys.2021.651977

**Published:** 2021-03-23

**Authors:** Marika Mikelsaar, Reet Mändar

**Affiliations:** Department of Microbiology, Institute of Biomedicine and Translational Medicine, University of Tartu, Tartu, Estonia

**Keywords:** gut microbiome, lactobacilli, probiotic, space flights, space missions

## Introduction

Recent years have shown a heightened interest in applying new technical achievements to improve the conditions for astronauts during space travels. However, there are still numerous shortcomings in the protection of space travelers' health concerning medical standards for space flight (Committee on Ethics Principles Guidelines for Health Standards for Long Duration Exploration Spaceflights et al., 2014). The paper of Turroni et al. (2020) has raised the important issue of maintaining the health and well-being of space travelers by supporting a well-functioning system of the host-microbiome in an extreme environment. Over the last 50 years, microgravity microbiome research has mainly focused on preventing gastrointestinal (GI) disturbances, as a consequence of dysbiosis due to various extreme space conditions (Gerassy-Vainberg et al., 2018; Jiang et al., 2019; Voorhies et al., 2019; Jones et al., 2020; Liu et al., 2020) and changed diets (Siddiqui et al., 2021). During space flight, dysbacteriosis with the prevalence of facultative pathogenic bacteria hampered travelers' well-being by causing inflammation and protein deficiency (Shilov et al., 1972). For the prevention of these disorders, the protective role of lactic acid bacteria in the GI tract was explored. The lactobiota of Soviet cosmonauts was investigated at the University of Tartu in Estonia, under the supervision of professor Akivo Lenzner (March 16, 1927–April 27, 2012)^1^ in a joint project with the Institute of Biomedical Problems of the Russian Academy of Sciences in Moscow, under the supervision of the research fellows V. M. Shilov and N. N. Lizko (Lentsner et al., 1973, 1981; Lencner et al., 1984).^1^

## Previous Findings and their Implications for Human Deep Space Explorations

From Baikonur (former USSR, now Kazakhstan) salivary and fecal samples from the cosmonauts and trainees from various expeditions like Soyuz and Salyut were sent to Tartu. In particular, the microbiota of every cosmonaut was studied, and the lactobacilli were isolated and identified. The subsequent results were highlighted in numerous papers written in Estonian, Russian, German, and English. Key findings of this research (Lencner et al., 1984) are listed in the following:

- The baseline counts of fecal lactobacilli were log_10_ 5.9 ± 1.3 CFU/g on MRS media;
- The composition of individual lactobiota varied across crewmembers and the most dominating species were *Lactobacillus acidophilus* and *Lactobacillus casei*;
- Compared to pre-flight, the counts of lactobacilli decreased for at least 2.0 log and that of lactic acid streptococci increased from log 4.9 ± 1.6 to 7.7 ± 0.8 CFU/g;
- Short duration flights (7 d Salut-6) seemed to have caused a decrease of dominant species of lactobacilli whereas some subordinate species of lactobacilli could be preserved;
- At the end of long duration space missions (75 and 185 d Salut-6) only the counts of *L. casei* decreased;
- Post-flight, in the rehabilitation period, the usually stable *L. acidophilus* was not found in some cosmonauts while the streptococci maintained high numbers.

## Discussion

Data of cosmonauts regarding the counts of lactobacilli are in line with data of healthy young terrestrial individuals (Haenel et al., 1957; Mikelsaar et al., 1972). The individuality of the microbiome, including the individuality of the lactobacilli composition, was first pointed out by the Japanese scientists Mitsuoka and Ohno (1977), and proved retrospectively with the data of 20 healthy Russian cosmonauts (Lencner et al., 1987; Mikelsaar and Mändar, 1993). At the start of short space missions (up to 1 week), the decrease from baseline values seemingly appeared to be due to the emotional stress in the extreme situations, as described previously (Holdeman et al., 1976). Some heterofermentative species of lactobacilli and lactic acid streptococci emerged in higher numbers. These discoveries have stayed valid and relevant until today. Contrary to short-term space flights, it seems that during long-term missions, changes in the composition of the lactobiota caused by the changed environment in space can be restored after a while. In Tartu, the isolated *Lactobacillus* strains were screened for antagonistic activity against potentially pathogenic bacteria and some other properties. Post flight, the rehabilitation period of these cosmonauts received special care to restore indigenous lactobiota. Due to the high inter-individual variability of the *Lactobacillus* species composition, first steps toward personalized medicine were already started in 80s. Chosen cosmonauts were provided with lyophilized cultures of their own beneficial *Lactobacillus sp*. strains. Three of these GI lactobacilli strains received the Russian patents in 1989 (Avtorskaja Spravka in Russian) for biological products.

Future deep space exploration missions should take the observed changes of the composition and functionality of the microbiome into account and a “well-fed” and healthy microbiome to support general well-being of crewmembers should be an important goal of dietary interventions. The value of the paper by Turroni et al. (2020) lays in renewing the ideas of probiotic strategies during long term space exploration missions. The authors have presented several probiotic products that help to protect various body functions. Recent studies have pointed toward an intricate relationship between the intestinal microbiota and the brain, forming the so called “gut-brain axis.” *Lactobacillus* strains have shown to produce neuroactive and neuroendocrine molecules to reduce stress-induced corticosterone and anxiety- and depression-related behavior (Bravo et al., 2011). However, the necessity for additional mechanistic studies under microgravity, including intervention studies and clinical trials are still necessary. On the contrary, the use of the individual's own protective bacteria seems a promising complementation for personalized medicine, and optimized healthcare for crewmembers. Considering usual adaptation times of the microbiome during space flight conditions, supplementation of capsulated lactobacilli would help to maintain crewmembers' gut health during the first weeks in the new space environment and during rehabilitation post-flight.

## Author Contributions

All authors listed have made a substantial, direct and intellectual contribution to the work, and approved it for publication.

## Conflict of Interest

The authors declare that the research was conducted in the absence of any commercial or financial relationships that could be construed as a potential conflict of interest.

## Footnotes

^1^Also written in the publications as Lencner and Lentsner.
